# Association of metabolic equivalent of task (MET) score in length of stay in hospital following radical cystectomy with urinary diversion: a multi-institutional study

**DOI:** 10.1007/s11255-021-02813-x

**Published:** 2021-03-06

**Authors:** Chun Shea, Abdul Rouf Khawaja, Khalid Sofi, Ghulam Nabi

**Affiliations:** 1 School of Medicine, University of Dundee, Dundee, Scotland UK; 2 Department of Urology, Sher-i-Kashmir Institute of Medical Sciences, Soura, Srinagar, India; 3 Department of Anesthesiology, Sher-i-Kashmir Institute of Medical Sciences, Soura, Srinagar, India; 4 Department of Urology, Ninewells Hospital, Dundee, Scotland UK

**Keywords:** Cystectomy, Radical cystectomy, Urinary diversion, Ileal conduit, MET score, Metabolic equivalent of task, Hospital stay length, Postoperative outcome, Postoperative complication

## Abstract

**Purpose:**

The Metabolic equivalent of task (MET) score is used in patients’ preoperative functional capacity assessment. It is commonly thought that patients with a higher MET score will have better postoperative outcomes than patients with a lower MET score. However, such a link remains the subject of debate and is yet unvalidated in major urological surgery. This study aimed to explore the association of patients’ MET score with their postoperative outcomes following radical cystectomy.

**Methods:**

We used records-linkage methodology with unique identifiers (Community Health Index/hospital number) and electronic databases to assess postoperative outcomes of patients who had underwent radical cystectomies between 2015 and 2020. The outcome measure was patients’ length of hospital stay. This was compared with multiple basic characteristics such as age, sex, MET score and comorbid conditions. A MET score of less than four (< 4) is taken as the threshold for a poor functional capacity. We conducted unadjusted and adjusted Cox regression analyses for time to discharge against MET score.

**Results:**

A total of 126 patients were included in the analysis. Mean age on date of operation was 66.2 (SD 12.2) years and 49 (38.9%) were female. A lower MET score was associated with a statistically significant lower time-dependent risk of hospital discharge (i.e. longer hospital stay) when adjusted for covariates (HR 0.224; 95% CI 0.077–0.652; *p* = 0.006). Older age (adjusted HR 0.531; 95% CI 0.332–0.848; *p* = 0.008) and postoperative complications (adjusted HR 0.503; 95% CI 0.323–0.848; *p* = 0.002) were also found to be associated with longer hospital stay. Other comorbid conditions, BMI, disease staging and 30-day all-cause mortality were statistically insignificant.

**Conclusion:**

A lower MET score in this cohort of patients was associated with a longer hospital stay length following radical cystectomy with urinary diversion.

**Supplementary Information:**

The online version contains supplementary material available at 10.1007/s11255-021-02813-x.

## Introduction

Major surgical procedures such as radical cystectomy with urinary diversion produce a large metabolic stress and inflammatory response [1]. The proinflammatory cytokine reaction in response to surgical injury may contribute to reducing patients’ postoperative quality of life [2]. Postoperative recovery course from major surgery affects patients’ quality of life, which is found to increase utilisation of health care resources [3]. For these reasons, predicting outcomes postoperatively becomes essential in major cancer resection associated with immediate complex reconstruction such as radical cystectomy with urinary diversion.

Physical activity energy expenditure is the most variable component of a person’s daily total energy expenditure [4] and is indirectly used to assess cardiopulmonary efficiency. The energy cost used for metabolic activity is expressed in metabolic equivalents. Metabolic equivalent of task (MET) is an established method of assessing energy cost for physical activities or exercise capacity [5]. A MET is defined as the amount of consumed oxygen at rest by a person, which is approximately 3.5 ml O_2_/kg/min [5, 6]. This can then be used to assess and quantify the functional capacity of an individual planned for major non-cardiac surgery [5]. The MET score is used to estimate preoperative exercise capacity of patients planned for major surgical procedures. It is a validated and recommended method of cardiovascular assessment [5, 7]. A MET score of 1 equates to limited activities such as eating and self-dressing; whereas, a score of 8 means that a person can do heavy work at home including scrubbing floors and moving furniture [8].

It is well established that MET score of less than four (< 4) indicates poor functional capacity [7] and is associated with higher perioperative morbidity and mortality [9, 10]. Poor physical activity is associated with poor perioperative outcomes [11, 12]. The functional physical activity of a patient is usually assessed by means of questionnaires or in-person interviews to allow a MET score estimate. This forms the core assessment of preoperative evaluation for major non-cardiac surgery [10].

In this study, we assessed the predictive ability of MET score in predicting the postoperative outcome of patients undergoing open radical cystectomy with urinary diversion. To the best of our knowledge, this aspect has not been reported in English literature. There are contradictory and inconclusive reports on the prediction of perioperative outcomes using cardiopulmonary exercise testing (CPET). Based on this strength of evidence, both American and European guidelines do not support any routine use of CPET in preoperative evaluation of non-cardiac surgery [7, 13]. Two cohort studies [14, 15] from the United Kingdom supported the use of CPET in preoperative assessment of radical cystectomy; however, a recent large meta-analysis [16] of non-cardiac surgical procedures including data from those two studies showed unknown benefits of preoperative CPET in major abdominal surgical procedures.

The present study is based on routinely collected data on MET score based on preoperative assessment and patient reporting of activities.

## Patients and methods

### Study cohort

We did a multi-institutional, international retrospective observational study consisting of all consecutive patients who opted and underwent open radical cystectomy with urinary diversion by ileal conduit for muscle invasive urinary bladder cancer between January 2015 and April 2020 at Ninewells Hospital, NHS Tayside, Scotland and Sher-i-Kashmir Institute of Medical Sciences (SKIMS), India. There were 126 patients included in our study; 100 patients were from Scotland and 26 patients from India. Two patients were excluded on the basis of missing data on any covariates or hospital length of stay. The study had institutional approval (Caldicott number IGTCAL7797 updated on 28/05/2020). Data collection was retrospective for all patients using electronic deterministic linkage methodology and unique identifier. The NHS Tayside health board is one of the fourteen Scottish Health authorities responsible for the delivery of healthcare in Scottish population. All inhabitants in the geographical location of Tayside are registered with the health authority and have a ten-digit Community Health Index (CHI) number as a unique identifier across all health records including primary and secondary care settings. Similarly, all patients at the Sher-i-Kashmir Institute of Medical Sciences, India, are assigned a unique hospital number. These unique identifiers were used to link data to the postoperative outcomes of all the patients in the study.

### Research method

We used retrospective records-linkage methodology using unique identifiers (Community Health Index/hospital number) and electronic databases to assess postoperative outcomes of patients who had underwent radical cystectomies at Ninewells Hospital, NHS Tayside and at Sher-i-Kashmir Institute of Medical Sciences, India between 2015 and 2020. The list of patients who underwent radical cystectomies was known and identified by their CHI number or hospital number, a unique identifier issued to every individual patient in NHS Scotland/or tertiary hospital in collaborating institute, then correspondingly deterministically linked to their existing records on hospital databases by the same CHI number/hospital number.

We derived patient MET scores from CHI/hospital number-linked preanaesthetic assessment records. A MET score of less than four (< 4) indicating poor functional capacity [7] was taken as the threshold for a poor functional capacity for our study. The MET score was determined as part of a routine preanaesthetic assessment in workup clinics in both institutions by questionnaire format. This was done by junior doctors supported by consultant anaesthetists and specialty registrars (middle grade doctors) in Scotland and experienced healthcare practitioners and consultant anaesthetists in India. Outcome measures was patients’ length of hospital stay; this was obtained from hospital discharge records. This was compared to multiple baseline demographic characteristics such as age, sex, BMI and comorbid conditions, which were obtained from electronically stored medical records. We conducted unadjusted and adjusted Cox regression analyses for time to discharge against MET score. Analyses were adjusted for baseline age, sex, comorbidities (hypertension, previous myocardial infarction, peripheral vascular disease, chronic obstructive pulmonary disease, diabetes mellitus, previous stroke/transient ischaemic attack, chronic kidney disease, hepatic failure), smoking status, body mass index (BMI), disease stage, postoperative complications and 30-day all-cause mortality. These factors were chosen as they were available in routinely collected preoperative assessment data and have been shown to affect perioperative and postoperative outcomes. Continuous variables were compared using Independent-Samples t-test for normally distributed variables. Categorical variables were compared using Chi-squared test. All analyses were conducted in SPSS v25 (IBM, New York, USA). A two-sided *p* value of < 0.05 was taken as significant for all analyses.

## Results

A total of 126 patients were included in the analysis. Mean age on date of surgical procedure was 66.2 (SD 12.2) years and 49 (38.9%) were female. Supplementary Table 1 shows the baseline characteristics of our cohort of patients. The characteristics of our Indian patients versus our Scottish patients are shown in Supplementary Table 2. We found that the Scottish patient proceeding to radical cystectomy was older, had a lower BMI, had more advanced disease, was more likely to experience postoperative complications but was less likely to experience mortality within 30 days postoperatively than their Indian counterparts. Supplementary Table 3 shows patients’ hospital stay length represented as a hazard ratio of risk of discharge over time, compared to their MET scores. A lower MET score was associated with a statistically significant lower time-dependent risk of hospital discharge (i.e. longer hospital stay) when adjusted for covariates (HR 0.224; 95% CI 0.077–0.652; *p* = 0.006). Older age (adjusted HR 0.531; 95% CI 0.332–0.848; *p* = 0.008) and postoperative complications (adjusted HR 0.503; 95% CI 0.323–0.848; *p* = 0.002) were also found to be associated with longer hospital stay. 30-day all-cause mortality, BMI, disease stage and other comorbid conditions were statistically insignificant.

There were 39 (31.0%) patients with Clavien–Dindo complications of grade 1 or 2 and 39 (31.0%) with complications of grade 3 or more shown in supplementary Table 4. A comprehensive breakdown of the most common complications is represented in supplementary Table 5. The tables of baseline characteristics of our cohort, Cox regression analyses and complication rates are available in the supplementary materials to this study.

## Discussion

In this analysis, a lower MET score was associated with a lower time-dependent risk of hospital discharge (i.e. higher risk of longer hospital stay) when adjusted with covariates. Furthermore, we also found postoperative complications and increased age were associated with a longer hospital stay length. The results stand to build on previous studies which showed a MET score of < 4 was associated with a higher perioperative morbidity and mortality [9, 10] and perioperative cardiovascular events [17, 18].

Mechanisms for a MET score being associated with a longer hospital stay in patients with radical cystectomy may be explained by the fact that any functional capacity reserve is essential to cope with huge metabolic stress and inflammatory response after significant trauma from a major surgery, particularly post-radical cystectomy [19]. Oxygen consumption following surgical stress is significantly increased and cardiopulmonary reserve is key to provide extra oxygen required to maintain tissue metabolism. The impact of major surgery on a patient’s physiological reserve can be understood through the role of surgery as a noxious stressor of the systemic inflammatory response syndrome (SIRS). In a major surgical procedure such as radical cystectomy, the incidence of SIRS is high; Wang et al. [1] in their study of surgical stress response in laparoscopic versus open radical cystectomy reported that the incidence of SIRS in their patients was 71.1%. SIRS on the molecular level leads to parenchymal cell damage with multiple organ dysfunction [19]. However, this does not manifest as organ dysfunction in the majority of patients due to their physiological reserves [19]. Rather, nowadays, a higher number of surgical patients of greater age with comorbid conditions are offered surgery; their reduced physiological reserves mean that any small alteration in organ function as a result of SIRS may lead to irreversible organ dysfunction [19].

Assessment of functional capacity or cardiopulmonary fitness remains the mainstay of preoperative workup for patients opting for major non-cardiac surgery. This invariably is based on a subjective assessment from taking history from patients. There are well-recognised limitations to the subjective assessments as described in this study. Wijeysundera et al. [20] in a prospective study reported a more objective method of assessing preoperative risk stratification for cardiac events and morbidity, relying on Duke Activity Status Index (DASI) questionnaires, CPET and NT pro-BNP testing rather than MET score alone to predict 30-day postoperative mortality or myocardial infarction. There are several differences between our observations and their published study. First, the population in the present study was drawn from a specific region in Scotland and India in contrast to Australia, Canada, New Zeeland and the United Kingdom reported by Wijeysundera et al. Second, the end point of study here is length of hospital stay. Third, our observation is procedure specific for radical surgery for muscle invasive urinary bladder cancer and reconstruction using segment of intestines; the group of patients in the reported study by Wijeysundera and colleagues was heterogeneous. Moreover, the questionnaire format of the DASI questionnaire involves assessment of complex physical activities which may only be self/patient-reported (e.g. have sexual relations, participate in strenuous sports like swimming, singles tennis football, basketball or skiing). The patient is not required to demonstrate competency of activities in the clinical environment to score. It must be noted that certain question items such as the question pertaining to “have sexual relations” are frequently missed by respondents [21]. Goldstein et al. [22] in a non-urological study of patients undergoing major head and neck surgery reported that the functional measures of the IADL and ADL (Instrumental activities of daily living and activities of daily living) scores were predictors of hospital length of stay. The limitations of both the IADL and ADL scoring are that they are not functional capacity specific; the IADL gives scoring for “Responsibility for Own Medications” and “Ability to Handle Finances”. These are tasks reflecting higher executive functions which is more of a reflection of cognitive function rather than functional capacity. Likewise, the ADL score concerns itself with a patient’s independence of activities of daily living, which, while there is an element of physical capacity to it, also concerns cognitive function. These measures of functional capacity may skew scoring as a patient with no cognitive impairment may score relatively highly while having poor physical capacity, and thus lack the physiological reserve for major surgery. It must also be noted that the IADL and ADL score of which tasks are assessed which the patient (or caregiver) self-reports which much like the DASI questionnaire introduces an element of subjectivity as the patient does not have to demonstrate such a functional task in the clinical environment to score.

In a mean age of cohort similar to ours, Prentis et al. [14] support cardiopulmonary exercise testing (CPET) as an independent predictor of postoperative complications and length of hospital stay. The authors report an anaerobic threshold (AT) of less than 12 ml/kg/min as a significant predictor of postoperative complications and delayed discharge [14]. One metabolic equivalent (MET) is the resting oxygen uptake in a sitting position and equals 3.5 ml/kg/min. A MET score of less than 4 is just roughly less than 14 ml/kg/ml, although in the absence of a more objective test, drawing a threshold has its own limitations.

Predicting postoperative complications is an appealing objective of surgical procedures planning including discussions with patients during counselling for a major surgical procedure. Although utilising CPET to assess cardiopulmonary reserve is standard, a carefully assessed and recorded MET score as in the present study provides some clues in predicting outcomes of radical cystectomy with urinary diversion. This may be one of the major reasons of CPET recommendations by the international Perioperative Exercise Testing and Training Society (POETTS) [23]. A careful assessment of preoperative cardiopulmonary reserve provides an opportunity to optimise cases and manage limited resources in places where their need is essential.

In our study, there were 39 (31.0%) patients with Clavien–Dindo complications of grade 1 or 2 and 39 (31.0%) with complications of grade 3 or more. This is compared to the cystectomy complication rates reported by Gontero et al. [24] (grade 1 or 2: 45.5%; grade 3 or more: 29.6%) and Paditar et al. [25] (grade 1 or 2: 71.8%; grade 3 or more: 28.0%). The variation between studies could be due to a smaller sample size of our cohort and a lack of a standardised reporting system for inclusion of complications.

Our study is the first of its kind to utilise routinely collected data of MET score with the comparison to the endpoint of hospital stay length for urological surgery. We were also able to work with a collaborating institute to increase our sample size.

One of the limitations of using the MET score to assess a person’s functional capacity is its intrinsic variations in describing or estimating the intensity of a physical activity for different people. As the MET score quantifies the amount of oxygen consumed at the basal metabolic rate for a person, it would be expected for a larger person to have an increased uptake of oxygen when compared to a smaller person due to an increased resting metabolic rate, thus a higher MET score for the same activity [6]. Likewise in people with the same mass but differing lean body masses, the patient with a higher lean body mass will have a higher MET score due to having a higher basal metabolic rate [6]. This means that inverse of people having the same MET score but different functional capacities holds true too for people of differing body masses or lean body masses.

Another limitation here would be the operator dependent nature of the MET score, as while each level of MET corresponds with an oxygen consumption level corresponding to a certain level of physical activity and capacity, the methodology of assessment of the MET score is not standardised between operators may be subject to variation. Moreover, with the MET score in a clinical environment, a patient’s history may form part of the determination of their MET score, thus there may be a subjective element in it, which leads on to the fact that we were unable to adjust for inter-operator variability retrospectively. We were furthermore unable to adjust for inter-institution variability in scoring as this is one of the inherent issues with working with retrospective observational data as well as multi-institutional data. One final limitation we note is that our cohort was limited in size (*n* = 126) due to the volume of patients undergoing radical cystectomies at both centres. Another limitation here would be the fact that our cohort of patients with < 4 MET score was limited to four (3%) of our patients.

Our results show that a MET score of less than four is associated with an increased length of stay; this warrants further investigation of the association of MET score with perioperative outcomes in radical cystectomy and raises the question of the appropriateness of including the MET score as part of a formal assessment of patients preoperatively. Further research should be based on a multicentre approach to further increase the sample size.

## Supplementary Information

Below is the link to the electronic supplementary material.

Supplementary file1 (DOCX 23 KB)
