# Supplementary material for: Association of metabolic equivalent of task (MET) score in length of stay in hospital following radical cystectomy with urinary diversion: a multi-institutional study
Source: Int Urol Nephrol. 2021 Mar 6;53(7):1305–10. doi: 10.1007/s11255-021-02813-x (PMC8192360; doi:10.1007/s11255-021-02813-x)
Supplement: Supplementary file 1 — Supplementary file1 (DOCX 23 KB) [file 11255_2021_2813_MOESM1_ESM.docx]

Table 1: Baseline characteristics of cohort

| Female (%) | 49.0 (38.9%) |
| --- | --- |
| Age (years) (SD) | 66.2 (12.2) |
| Age >65 (%) | 73.0 (57.9%) |
| MET score mean (SD) | 6.5 (1.5) |
| MET score <4 (%) | 4.0 (3.2%) |
| Post-operative complications (%) | 78.0 (61.9%) |
| Myocardial infarction (%) | 12.0 (9.5%) |
| Hypertension (%) | 74.0 (58.7%) |
| Peripheral vascular disease (%) | 13.0 (10.3%) |
| Smoking (%) | 66.0 (52.4%) |
| COPD (%) | 10.0 (7.9%) |
| Diabetes mellitus (%) | 18.0 (14.3%) |
| Stroke/TIA (%) | 7.0 (5.6%) |
| Renal failure CKD ≥4 (%) | 17.0 (13.5%) |
| Hepatic failure (%) | 1.0 (0.8%) |
| BMI ≥25kg/m^2^ | 99.0 (78.6%) |
| Disease stage (≥pT3 or any nodal disease) (%) | 66.0 (52.4%) |
| 30-day all-cause mortality (%) | 3 (2.4%) |

Table 2: Baseline characteristics of Scottish cohort versus Indian cohort

|  | Ninewells, Scotland (n=100) | SKIMS, India  (n=26) | P value |
| --- | --- | --- | --- |
| Female (%) | 41.0 (41.0) | 9.0 (34.6) | 0.62 |
| Age (years) (SD) | 69.6 (9.0) | 52.9 (14.1) | <0.001 |
| Age >65 (%) | 69.0 (69.0) | 3 (11.5) | <0.001 |
| MET score (SD) | 6.6 (1.4) | 5.9 (1.5) | 0.05 |
| MET score <4 (%) | 4.0 (4) | 0 (0) | 0.30 |
| Post-operative complications (%) | 76.0 (76.0) | 2.0 (7.7) | <0.001 |
| Myocardial infarction (%) | 59.0 (59.0) | 15.0 (57.7) | 0.72 |
| Hypertension (%) | 10.0 (10.0) | 2.0 (7.7) | 0.90 |
| Peripheral vascular disease (%) | 13.0 (13.0) | 0 (0) | 0.05 |
| Smoking (%) | 54.0 (54.0) | 12.0 (46.2) | 0.48 |
| COPD (%) | 8.0 (8.0) | 2.0 (7.7) | 0.96 |
| Diabetes mellitus (%) | 16.0 (16.0) | 2.0 (7.7) | 0.28 |
| Stroke/TIA (%) | 5.0 (5.0) | 2.0 (7.7) | 0.59 |
| Renal failure CKD ≥4 (%) | 17.0 (17.0) | 0 (0) | 0.024 |
| Hepatic failure (%) | 1.0 (1.0) | 0 (0) | 0.61 |
| BMI (kg/m^2^) (SD) | 27.4 (5.6) | 31.4 (3.5) | 0.001 |
| BMI ≥25kg/m^2^ (%) | 74.0 (74.0) | 26.0 (100.0) | 0.003 |
| Disease stage (≥pT3 or any nodal disease) (%) | 56.0 (56.0) | 9.0 (34.6) | 0.042 |
| 30-day all-cause mortality (%) | 1.0 (1.0) | 2.0 (7.7) | 0.046 |

Table 3: Cox-regression analyses of MET score <4 to outcome measure of hospital stay length, adjusted and unadjusted for covariates

| MET4 | Hazard ratio (95% Confidence interval) | P value |
| --- | --- | --- |
| ***Unadjusted analyses*** |  |  |
| MET score <4 | 0.409 (0.150-1.119) | 0.08 |
| ***Adjusted analyses*** |  |  |
| MET score <4 | 0.224 (0.077-0.652) | 0.006 |
| Female | 0.849 (0.541-1.332) | 0.48 |
| Age >65 | 0.531 (0.332-0.848) | 0.008 |
| Post-operative complications | 0.503 (0.323-0.848) | 0.002 |
| Hypertension | 1.559 (0.996-2.439) | 0.05 |
| Myocardial infarction | 0.979 (0.478-2.002) | 0.95 |
| Peripheral vascular disease | 1.627 (0.773-3.423) | 0.20 |
| Smoking | 1.118 (0.743-1.682) | 0.59 |
| COPD | 0.681 (0.313-1.479) | 0.33 |
| Diabetes mellitus | 1.264 (0.691-2.312) | 0.45 |
| Stroke/TIA | 0.828 (0.326-2.102) | 0.69 |
| Renal failure CKD ≥4 | 0.772 (0.427-1.395) | 0.39 |
| Hepatic failure | 1.171 (0.126-10.841) | 0.89 |
| BMI ≥25kg/m^2^ | 1.104 (0.685-1.779) | 0.69 |
| Disease stage (≥pT3 or any nodal disease) | 1.107 (0.715-1.713) | 0.65 |
| 30-day all-cause mortality | 0.00 (0.00-<0.001) | 0.95 |

Table 4: Dindo-Clavien classification of post-operative complications in cohort

| Dindo-Clavien classification | Number of patients | Percentage of patients (%) |
| --- | --- | --- |
| None | 48 | 38.1 |
| I | 11 | 8.7 |
| II | 28 | 22.2 |
| III | 15 | 11.9 |
| IV | 21 | 16.7 |
| V | 3 | 2.4 |

Table 5: Breakdown of top 10 most common post-operative complications in cohort

| Complication | Number of patients | Percentage of patients (%) |
| --- | --- | --- |
| Sepsis | 18 | 14.3 |
| Intraabdominal/pelvic collections | 13 | 10.3 |
| Anaemia requiring transfusion | 11 | 8.7 |
| Acute kidney injury | 9 | 7.1 |
| Wound dehiscence | 9 | 7.1 |
| Atrial fibrillation/flutter | 8 | 6.4 |
| Hospital acquired pneumonia | 6 | 4.8 |
| Ileus | 6 | 4.8 |
| Wound infection | 6 | 4.8 |
| Small bowel obstruction | 6 | 4.8 |
